# Extracellular phosphate enhances the function of F508del-CFTR rescued by CFTR correctors

**DOI:** 10.1016/j.jcf.2021.04.013

**Published:** 2021-09

**Authors:** Vinciane Saint-Criq, Yiting Wang, Livia Delpiano, JinHeng Lin, David N. Sheppard, Michael A. Gray

**Affiliations:** a Biosciences Institute, Faculty of Medical Sciences, Newcastle University, Newcastle upon Tyne, United Kingdom; b School of Physiology, Pharmacology and Neuroscience, University of Bristol, Biomedical Sciences Building, University Walk, Bristol, United Kingdom; c Université Paris-Saclay, INRAE, AgroParisTech, Micalis Institute, 78350, Jouy-en-Josas, France

**Keywords:** F508del-CFTR, CFTR correctors, Elexacaftor-tezacaftor-ivacaftor (Trikafta), Airway epithelia, Phosphate, SLC34A2

## Abstract

*Background*: The clinical response to cystic fibrosis transmembrane conductance regulator (CFTR) modulators varies between people with cystic fibrosis (CF) of the same genotype, in part through the action of solute carriers encoded by modifier genes. Here, we investigate whether phosphate transport by SLC34A2 modulates the function of F508del-CFTR after its rescue by CFTR correctors.

*Methods*: With Fischer rat thyroid (FRT) cells heterologously expressing wild-type and F508del-CFTR and fully-differentiated CF and non-CF human airway epithelial cells, we studied SLC34A2 expression and the effects of phosphate on CFTR-mediated transepithelial ion transport. F508del-CFTR was trafficked to the plasma membrane by incubation with different CFTR correctors (alone or in combination) or by low temperature.

*Results*: Quantitative RT-PCR demonstrated that both FRT and primary airway epithelial cells express SLC34A2 mRNA and no differences were found between cells expressing wild-type and F508del-CFTR. For both heterologously expressed and native F508del-CFTR rescued by either VX-809 or C18, the magnitude of CFTR-mediated Cl^−^ currents was dependent on the presence of extracellular phosphate. However, this effect of phosphate was not detected with wild-type and low temperature-rescued F508del-CFTR Cl^−^ currents. Importantly, the modulatory effect of phosphate was observed in native CF airway cells exposed to VX-445, VX-661 and VX-770 (Trikafta) and was dependent on the presence of both sodium and phosphate.

*Conclusions*: Extracellular phosphate modulates the magnitude of CFTR-mediated Cl^−^ currents after F508del-CFTR rescue by clinically-approved CFTR correctors. This effect likely involves electrogenic phosphate transport by SLC34A2. It might contribute to inter-individual variability in the clinical response to CFTR correctors.

## Highlights

• CFTR correctors rescue the plasma membrane expression of F508del-CFTR.
• Extracellular phosphate enhances F508del-CFTR function rescued by CFTR correctors.
• Cystic fibrosis airway epithelia express the phosphate transporter SLC34A2.
• Extracellular phosphate levels might contribute to variable drug responses.

## 1 Introduction

The genetic disease cystic fibrosis (CF) is caused by mutations in the epithelial anion channel cystic fibrosis transmembrane conductance regulator (CFTR) [1,2]. Based on the mechanisms of CFTR dysfunction [1,2], CFTR modulators have been developed to rescue the plasma membrane expression, stability and function of CF mutants. Just as disease severity varies between people with CF harbouring the same mutations [3,4], so to do responses to CFTR modulators [5], [6], [7], [8]. This variability is thought to be related to environmental factors and polymorphisms in modifier genes [5]. For example, two recent studies demonstrated that variability in the response to the CFTR corrector lumacaftor [9] was due to inter-patient, rather than intra-patient, differences when CFTR function was assessed *in vitro* after correction [6,7]. Additionally, exonic variants may also impact the efficacy of CFTR correction [8]. To assist the development of personalised therapies for CF, these and other studies have led to the classification of mutations based on their response to CFTR modulators, known as theratyping [2]. It is therefore crucial to identify and understand the molecular players that influence the response to CFTR modulators.

Genome Wide Association Studies (GWAS) and *in vitro* functional investigations have identified several modifier genes that affect CF disease severity and/or the response to CFTR modulators [10], [11], [12], [13], [14]. Interestingly, three of the identified modifier genes, SLC6A14, SLC9A3 and SLC26A9, are members of the Solute Carrier (SLC) transporter superfamily (https://www.bioparadigms.org/). Although understanding of why polymorphisms in SLC transporter genes have modulatory effects is incomplete, recent work has provided important insights for SLC6A14, an electrogenic, neutral and cationic amino acid transporter found at the apical membrane of airway and intestinal epithelial cells. L-Arginine transport by SLC6A14 modulated bacterial attachment to airway epithelia, CFTR activity and the response of F508del-CFTR to CFTR modulators [15], [16], [17], [18]. These data suggest that targeting the L-arginine pathway might be beneficial to CF patients treated with CFTR modulators.

We speculated that other SLC transporters might influence CFTR activity and/or the response of CF mutants to CFTR modulators. We were intrigued to learn that mutations in the *SLC34A2* gene, which encodes an apically–located Na^+^-dependent phosphate transporter, were linked to pulmonary alveolar microlithiasis [19], as a consequence of SLC34A2 transporter dysfunction [20,21]. This work identified an important role for SLC34A2 to remove phosphate from airway surface liquid (ASL). Since cytosolic phosphate levels influence CFTR function [22], we hypothesised that phosphate might be an unrecognised parameter governing the response to CFTR modulators. To investigate this possibility, we tested the effects of extracellular phosphate on CFTR-mediated Cl^−^ currents in Fischer rat thyroid (FRT) epithelia heterologously expressing F508del-CFTR and fully differentiated human airway epithelial cells (genotype: F508del/F508del), following rescue of F508del-CFTR with CFTR correctors (lumacaftor [VX-809], C18 or elexacaftor [VX-445] plus tezacaftor [VX-661] with the CFTR potentiator ivacaftor [VX-770]; Trikafta) or low temperature. Our results demonstrated that rescued F508del-CFTR activity was markedly dependent on extracellular phosphate, but this was not the case for either low temperature-rescued F508del-CFTR or wild-type CFTR. These data demonstrate that luminal phosphate has a hitherto unknown stimulatory effect on CFTR corrector-rescued F508del-CFTR and suggest that phosphate has the potential to impact the outcome of CFTR corrector therapies.

## 2 Materials and methods

### 2.1 Cells and cell culture

Primary non-CF and CF (genotype: F580del/F508del) human Airway Epithelial Cells (hAECs) and Fischer Rat Thyroid (FRT) epithelial cells heterologously expressing wild-type (WT) and F508del-CFTR were supplied, cultured and used as described in the Supplementary material.

### 2.2 RNA extraction, PCR and real-time quantitative PCR analysis

Total RNA extraction and real-time quantitative PCR (qPCR) were performed as described in the Supplementary material.

### 2.3 Short‐circuit current measurements

The Ussing chamber technique was used to record the transepithelial resistance (R_t_) and short-circuit current (I_sc_) due to CFTR-mediated transepithelial anion transport as described in the Supplementary material. CFTR was activated with 10 μM forskolin, potentiated by 10 μM P5 (ΔF508_act_-02) and inhibited with CFTR_inh_-172 (I172, 20 μM). To traffic F508del-CFTR to the plasma membrane, cells were pre-treated at 37°C with (i) either VX-809 (3 μM) or C18 (3 μM) for 48 h, or (ii) a combination of VX-445 (2 µM), VX-661 (3 µM) and VX-770 (1 µM) for 24 h before mounting in Ussing chambers; the vehicle was DMSO (0.06% - 0.1% v·v^‐1^). In other experiments, F508del-CFTR was trafficked to the plasma membrane by growing cells at 27°C for 48 h.

### 2.4 Statistical analysis

Results are expressed as means ± SD of n observations with statistical analyses performed as described in the Supplementary material.

## 3 Results

### 3.1 The phosphate transporter SLC34A2 is expressed in primary cultures of human airway epithelial cells

**Fig. 1:**
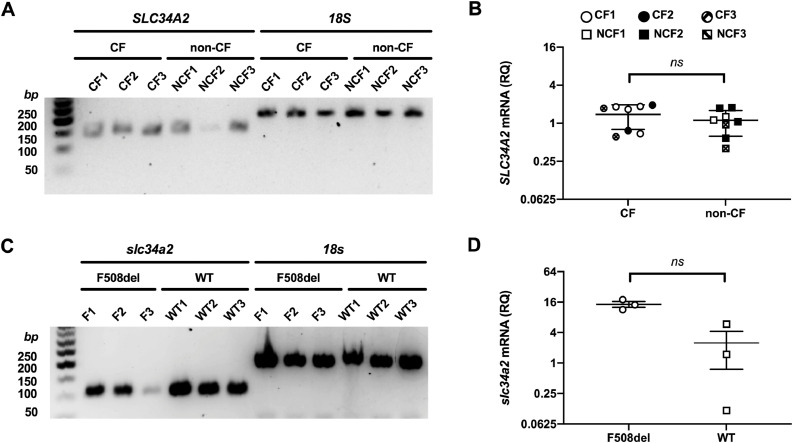
Messenger RNA expression of the phosphate transporter SLC34A2 in hAECs and FRT cells. (**A** and **C**) Agarose gel electrophoresis (2% agarose) of PCR amplified products using specific primer pairs for (**A**) *SLC34A2* and *18S* rRNA with bands at 155 and 209 bp, respectively, and (**C**) *slc34a2* and *18s* rRNA with bands at 109 and 208 bp, respectively. (**B** and **D**) Relative quantity (RQ) of mRNA for (**B**) *SLC34A2* in primary cultures of hAECs from CF and non-CF (NCF) donors and (**D**) *slc34a2* in F508del- and wild-type CFTR FRT cells. Symbols represent individual values and lines are means ± SD (**B**, n = 8 from 3 donors each; P = 0.33; **D**, n = 3; P = 0.1; Mann-Whitney rank sum test).

The Human Protein Atlas (https://www.proteinatlas.org/ENSG00000157765-SLC34A2/tissue) indicates that SLC34A2 (Na^+^-dependent phosphate cotransporter 2B; NaPi-2B) mRNA and protein are found in the lung, including prominent expression in alveolar type II cells. To learn whether SLC34A2 is expressed in respiratory airway epithelial cells, we studied primary cultures of CF (genotype: F508del/F508del) and non-CF airway epithelial cells. Fig. 1A demonstrates that SLC34A2 was expressed in CF and non-CF human airway epithelial cells, while Fig. 1B reveals that the relative levels of SLC34A2 mRNA did not differ between CF and non-CF human airway epithelial cells. Fig. 1C and D demonstrate that FRT cells express rat slc34a2 and its expression did not differ between FRT cells heterologously expressing wild-type and F508del-CFTR. These data suggest that FRT epithelia heterologously expressing human CFTR might be used to examine the effects of SLC34A2-mediated phosphate transport on CFTR function.

### 3.2 Phosphate enhances the function of F508del-CFTR rescued by CFTR correctors

**Fig. 2:**
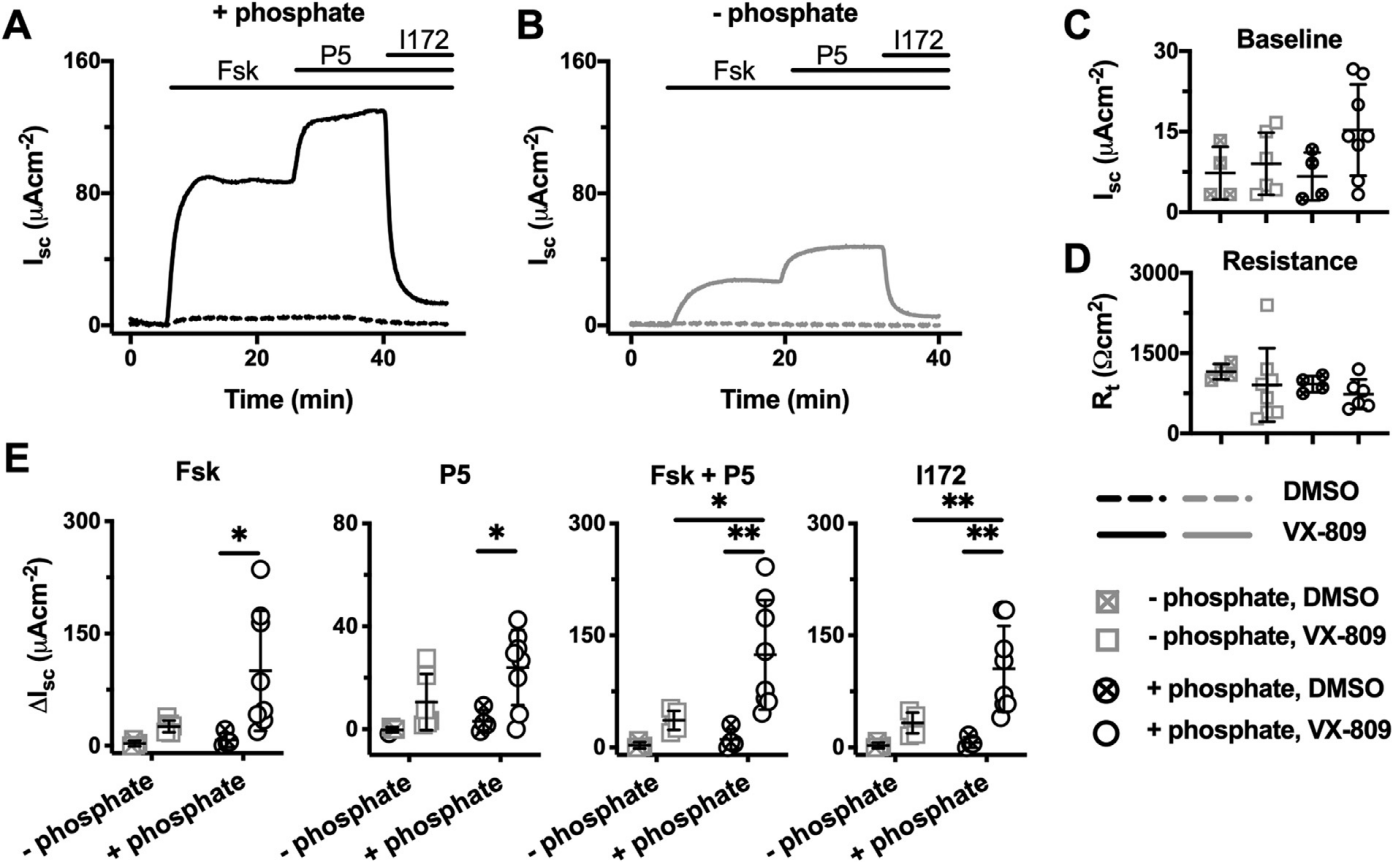
Phosphate increases lumacaftor-rescued F508del-CFTR-mediated Cl^−^ currents in FRT epithelia. (**A** and **B**) Representative I_sc_ recordings of lumacaftor-rescued F508del-CFTR in the presence (**A**) and absence (**B**) of phosphate (1.24 mM K_2_HPO_4_ and 2.4 mM KH_2_PO_4_) in the Krebs Ringer Buffer (KRB). Prior to study, F508del-CFTR-expressing FRT epithelia were treated with lumacaftor (VX-809; 3 μM) or DMSO (0.1% v·v^−1^) for 48 h at 37°C. At the indicated times, F508del-CFTR-mediated Cl^−^ currents were activated with forskolin (Fsk; 10 μM), potentiated with P5 (10 μM) and inhibited with CFTR_inh_-172 (I172; 20 μM); continuous lines indicate the presence of compounds in the apical solution only or the apical and basolateral solutions (forskolin) during I_sc_ recordings. Data were normalised by subtraction of the baseline current prior to F508del-CFTR activation by forskolin. (**C** – **E**) Summary data show the magnitude of baseline I_sc_, R_t_ before forskolin addition and the change in I_sc_ (ΔI_sc_) for the indicated conditions. Symbols represent individual values and lines are means ± SD (VX-809: +phosphate, n = 8; –phosphate, n = 6; DMSO: n = 4); *, P < 0.05; **, P < 0.01 (Two-way ANOVA with Tukey's multiple comparison test).

To investigate whether extracellular phosphate modulates the activity of F508del-CFTR after its rescue by CFTR correctors, FRT epithelia heterologously expressing F508del-CFTR were treated with either lumacaftor (VX-809; 3 μM) or vehicle (DMSO 0.1% v·v^‐1^) for 48 h at 37°C before mounting in Ussing chambers. To maximise CFTR-dependent currents, FRT epithelia were bathed in a basolateral to apical Cl^−^ gradient ([Cl^−^]_basolateral_, 119.8 mM; [Cl^−^]_apical_, 6 mM) in the absence or presence of phosphate (1.24 mM K_2_HPO_4_ and 2.4 mM KH_2_PO_4_) (Fig. 2A and B). Under these conditions, there was no significant difference in resting short-circuit current (I_sc_) or transepithelial electrical resistance (R_t_) between the four different conditions (P > 0.05) (Fig. 2C and D). As expected, when compared to the DMSO controls, F508del-CFTR-expressing FRT epithelia that were pre-treated with VX-809, displayed markedly larger responses to the cAMP agonist, forskolin (Fsk, 10 μM) and the CFTR potentiator P5 (5 μM), which were fully blocked by the CFTR inhibitor, CFTR_inh_-172 (I172, 20 μM) (Fig. 2A, B and E), confirming they were the result of F508del-CFTR that had trafficked to the apical membrane in response to VX-809 [9]. Strikingly, the magnitude of CFTR-mediated Cl^−^ current generated by F508del-CFTR-expressing FRT epithelia pre-treated with VX-809 was markedly reduced when phosphate was absent from the recording solutions (Fig. 2A, B and E). Indeed, CFTR_inh_-172-sensitive currents were reduced ~4-fold by the absence of phosphate.

**Fig. 3:**
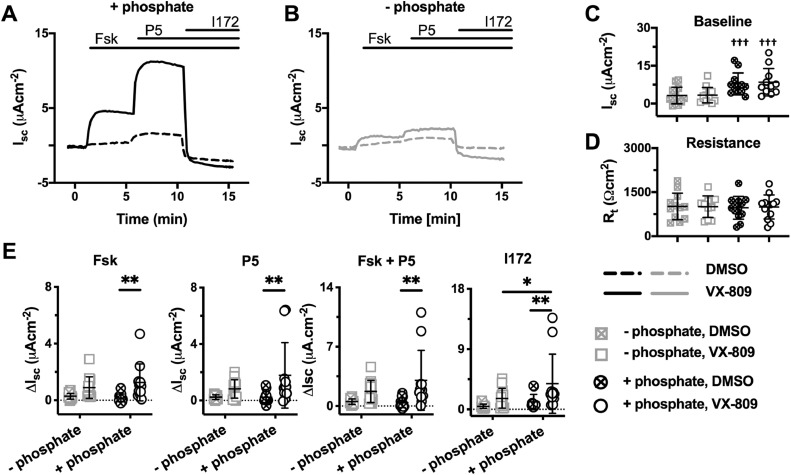
Phosphate enhances lumacaftor-rescued F508del-CFTR-mediated Cl^−^ currents in hAEC epithelia. (**A** and **B**) Representative I_sc_ recordings of lumacaftor-rescued F508del-CFTR in the presence (**A**) and absence (**B**) of phosphate (1.24 mM K_2_HPO_4_ and 2.4 mM KH_2_PO_4_) in the KRB. Prior to study, hAEC epithelia (genotype: F508del/F508del) were treated with lumacaftor (VX-809; 3 μM) or DMSO (0.1% v·v^−1^) for 48 h at 37°C. At the indicated times, F508del-CFTR-mediated Cl^−^ currents were activated with forskolin (Fsk; 10 μM), potentiated with P5 (10 μM) and inhibited with CFTR_inh_-172 (I172; 20 μM); continuous lines indicate the presence of compounds in the apical solution only or the apical and basolateral solutions (forskolin) during I_sc_ recordings. Experiments were performed in the presence of amiloride (10 μM) in the apical solution. Data were normalised by subtraction of the steady-state current after amiloride addition prior to F508del-CFTR activation by forskolin. (**C** – **E**) Summary data show the magnitude of baseline I_sc_ and R_t_ before amiloride addition and the change in I_sc_ (ΔI_sc_) for the indicated conditions. Symbols represent individual values and lines are means ± SD (VX-809: +phosphate, n = 9; –phosphate, n = 11; DMSO: +phosphate, n = 10; –phosphate, n = 11); *, P < 0.05; **, P < 0.01; ^†††^, P < 0.001 (Repeated Measure two-way ANOVA with Sidak's multiple comparison test).

To test whether this dependency on extracellular phosphate was also observed in epithelial cells that endogenously express F508del-CFTR, experiments were repeated using fully differentiated epithelia of CF hAECs homozygous for F508del-CFTR (Fig. 3). In contrast to F508del-CFTR-expressing FRT epithelia (Fig. 2C), baseline I_sc_ was higher in the presence of phosphate (Fig. 3C), but R_t_ was not affected (Fig. 3D). Importantly, similar to F508del-CFTR-expressing FRT epithelia, extracellular phosphate markedly improved the response of VX-809-rescued F508del-CFTR to forskolin and P5. Indeed, the resulting CFTR_inh_-172-sensitive currents were ~2.5-fold greater than those measured in CF hAEC epithelia pre-treated with VX-809 studied in the absence of phosphate (Fig. 3A, B and E), confirming the results obtained with F508del-CFTR-expressing FRT epithelia (Fig. 2).

To learn whether the response to phosphate is specific to VX-809, experiments were repeated using both cell models with the CFTR corrector, C18 an analogue of VX-809 which produces comparable levels of F508del-CFTR correction to VX-809 [23]. Overall, we observed a similar effect of extracellular phosphate on the response to forskolin and P5 in the C18-pre-treated F508del-CFTR-expressing FRT epithelia (Supplementary Fig. 1) and the C18-pre-treated CF hAEC epithelia (Supplementary Fig. 2), to that found with VX-809-pre-treated epithelia. Thus, extracellular phosphate enhances F508del-CFTR function rescued by the CFTR correctors VX-809 and C18.

### 3.3 Phosphate fails to enhance the function of F508del-CFTR after correction by low temperature incubation

We also investigated whether the response to phosphate was evident using low temperature to rescue F508del-CFTR. For these experiments, F508del-CFTR-expressing FRT epithelia were incubated at 27°C for 48 h to traffic F508del-CFTR to the apical membrane [24] before epithelia were mounted in Ussing chambers to measure I_sc_ at 37°C. Supplementary Fig. 3A shows representative I_sc_ traces of rescued F508del-CFTR-expressing FRT epithelia in the absence and presence of phosphate. The presence of phosphate did not affect baseline I_sc_ nor R_t_ (Supplementary Fig. 3B and C), but, in marked contrast to VX-809- and C18-treated F508del-CFTR-expressing FRT epithelia (Fig. 2 and Supplementary Fig. 1), there was no effect of phosphate on the magnitude of the forskolin-stimulated and P5-potentiated I_sc_, nor the CFTR_inh_-172-sensitive I_sc_ (Supplementary Fig. 3D). As a result, when compared to VX-809- and C18-corrected F508del-CFTR, low temperature-rescued F508del-CFTR showed a much larger forskolin-stimulated and P5-potentiated I_sc_ in the absence of phosphate (~4-fold greater) (Fig. 2E and Supplementary Figs. 1E and 3D). This result suggests that low temperature rescue eliminated the ability of extracellular phosphate to modulate F508del-CFTR activity.

To learn whether the effect of extracellular phosphate was restricted to misfolded/mutant CFTR, we studied wild-type CFTR. Supplementary Fig. 4 demonstrates that phosphate was without effect on wild-type CFTR heterologously expressed in FRT epithelia. Extracellular phosphate had no effect on baseline I_sc_, R_t,_ the forskolin-stimulated P5-potentiated I_sc_ nor the CFTR_inh_-172-sensitive I_sc_ (Supplemental Fig. 4). Similarly, Supplementary Fig. 5 reveals that phosphate was without effect on native CFTR in non-CF hAEC epithelia. Taken together, these data suggest that the ability of extracellular phosphate to enhance F508del-CFTR function only occurs in cells where mutant CFTR is rescued with CFTR correctors.

### 3.4 Phosphate enhances the function of F508del-CFTR rescued by elexacaftor-tezacaftor-ivacaftor

**Fig. 4:**
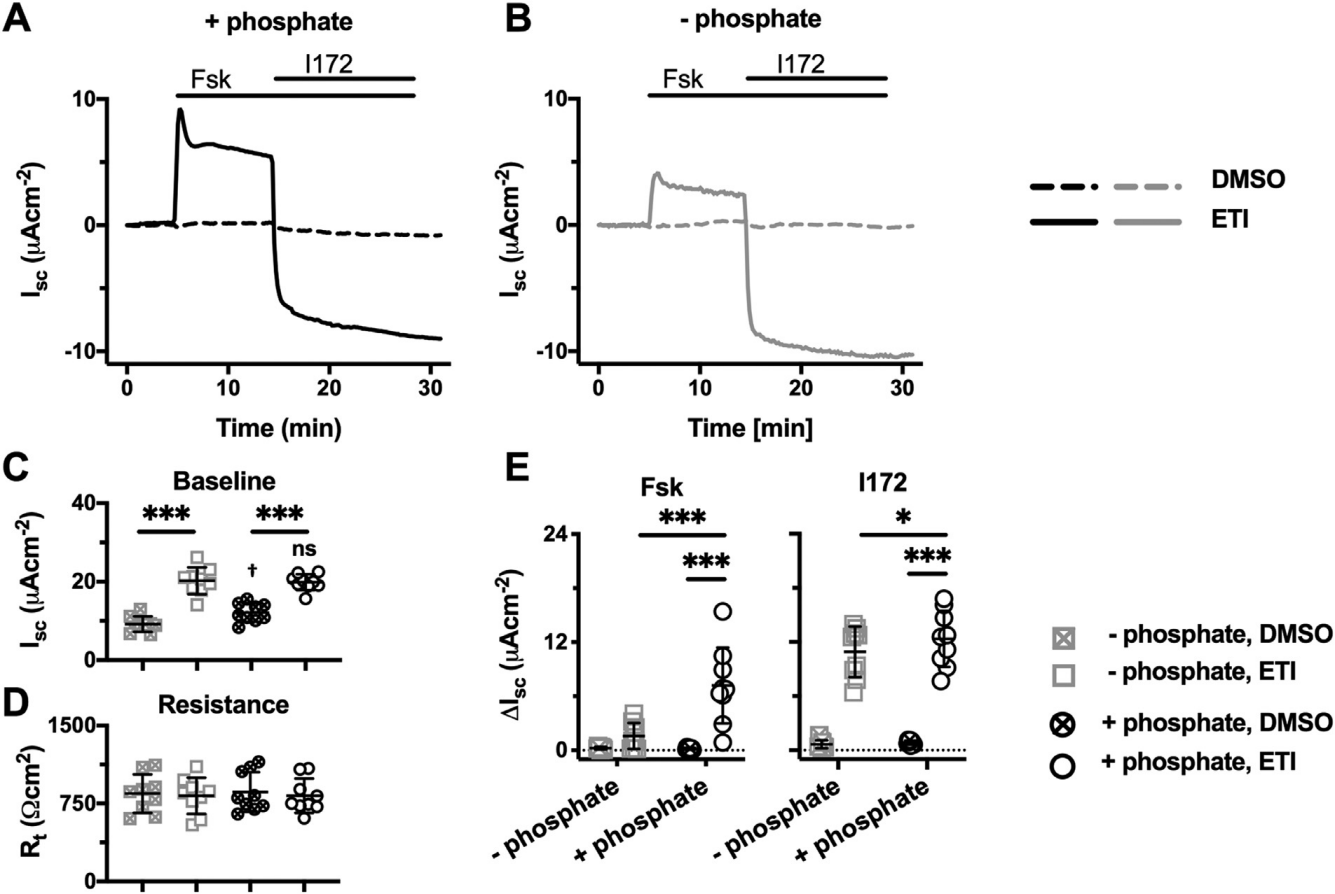
Phosphate enhances elexacaftor-tezacaftor-ivacaftor-rescued F508del-CFTR-mediated Cl^−^ currents in hAEC epithelia. (**A** and **B**) Representative I_sc_ recordings of elexacaftor-tezacaftor-ivacaftor (ETI)-rescued F508del-CFTR in the presence (**A**) and absence (**B**) of phosphate (1.24 mM K_2_HPO_4_ and 2.4 mM KH_2_PO_4_) in the KRB. Prior to study, hAEC epithelia (genotype: F508del/F508del) were treated with VX-445 (2 µM), VX-661 (3 µM) and VX-770 (1 µM) or DMSO (0.06% v·v^−1^) for 24 h at 37°C. At the indicated times, F508del-CFTR-mediated Cl^−^ currents were activated with forskolin (Fsk; 10 μM) and inhibited with CFTR_inh_-172 (I172; 20 μM); continuous lines indicate the presence of compounds in the apical solution only, or the apical and basolateral solutions (forskolin) during I_sc_ recordings. Experiments were performed in the presence of amiloride (10 μM) in the apical solution. Data were normalised by subtraction of the steady-state current after amiloride addition prior to F508del-CFTR activation by forskolin. (**C** – **E**) Summary data show the magnitude of baseline I_sc_ and R_t_ before amiloride addition and the change in I_sc_ (ΔI_sc_) for the indicated conditions. Symbols represent individual values and lines are means ± SD (elexacaftor-tezacaftor-ivacaftor (ETI): +phosphate, n = 9; –phosphate, n = 9; DMSO: +phosphate, n = 9; –phosphate, n = 9); * P < 0.05; ***, P < 0.001; ^†^, P < 0.05 vs. –phosphate (Two-way ANOVA with Sidak's multiple comparison test).

In 2019, elexacaftor-tezacaftor-ivacaftor (Trikafta), the combination of two CFTR correctors, VX-445 (elexacaftor) and VX-661 (tezacaftor) with the CFTR potentiator VX-770 (ivacaftor), was approved for clinical use in people with CF carrying the F508del mutation on at least one allele (https://www.fda.gov/news-events/press-announcements/fda-approves-new-breakthrough-therapy-cystic-fibrosis), following very positive clinical trial results [25,26]. *In vitro* studies demonstrate that VX-445 synergistically rescued F508del-CFTR processing when used in combination with VX-661, and that the triple combination of VX-445, VX-661 and VX-770 restored ~ 62% of wild-type CFTR function to nasal epithelia expressing native F508del-CFTR [27]. To learn whether the effect of extracellular phosphate was also observed when F508del-CFTR was rescued by elexacaftor-tezacaftor-ivacaftor (ETI), fully differentiated epithelia of CF hAECs homozygous for F508del-CFTR were incubated at 37°C for 24 h with the triple drug combination [27], before epithelia were mounted in Ussing chambers (Fig. 4). Pre-treatment with elexacaftor-tezacaftor-ivacaftor caused a striking increase in baseline I_sc_, which was not dependent on phosphate (Fig. 4C), but R_t_ was unaffected (Fig. 4D). Importantly, the response to forskolin was noticeably improved in the presence of extracellular phosphate in elexacaftor-tezacaftor-ivacaftor rescued F508del-CFTR epithelia, which was mirrored in the magnitude of the CFTR_inh_-172-sensitive current (Fig. 4E), consistent with the results obtained with VX-809 (Fig. 3). However, unlike epithelia pre-treated with VX-809, the results in Fig. 4, demonstrate that elexacaftor-tezacaftor-ivacaftor caused a substantial increase in corrected F508del-CFTR function in the absence of forskolin stimulation, consistent with previous results [27].

### 3.5 Sodium-dependence of the enhancement of elexacaftor-tezacaftor-ivacaftor -rescued F508del-CFTR function by phosphate

**Fig. 5:**
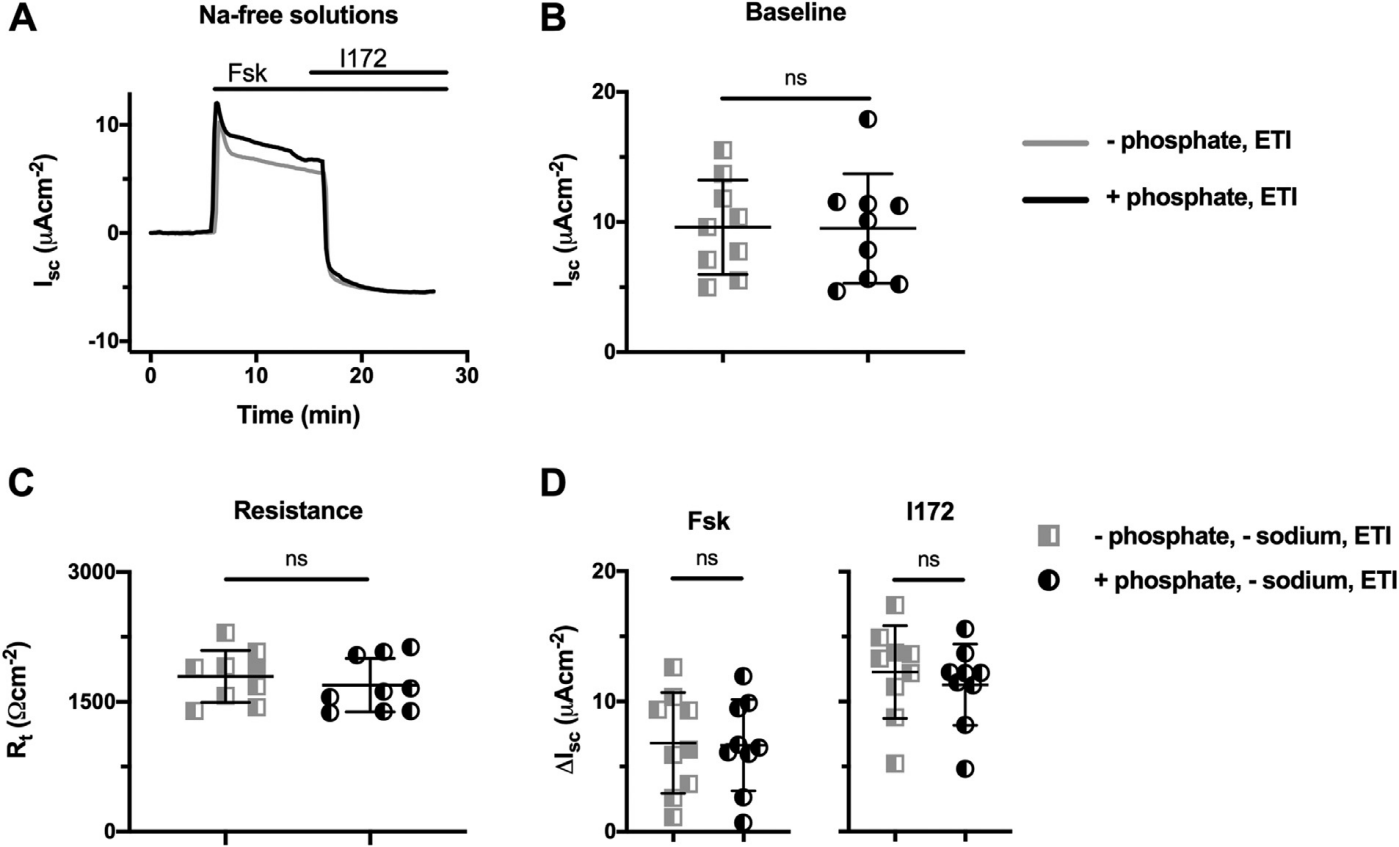
The enhancement by phosphate of F508del-CFTR function in hAEC epithelia after rescue by elexacaftor-tezacaftor-ivacaftor is dependent on external sodium. (**A**) Representative I_sc_ recordings of elexacaftor-tezacaftor-ivacaftor (ETI)-rescued F508del-CFTR in the absence or presence of phosphate (1.24 mM K_2_HPO_4_ and 2.4 mM KH_2_PO_4_) using a sodium-free KRB. Prior to study, hAEC epithelia (genotype: F508del/F508del) were treated with VX-445 (2 µM), VX-661 (3 µM) and VX-770 (1 µM) or DMSO (0.06% v·v^−1^) for 24 h at 37°C. At the indicated times, F508del-CFTR-mediated Cl^−^ currents were activated with forskolin (Fsk; 10 μM) and inhibited with CFTR_inh_-172 (I172; 20 μM); continuous lines indicate the presence of compounds in the apical solution only, or the apical and basolateral solutions (forskolin) during I_sc_ recordings. Experiments were performed in the presence of amiloride (10 μM) in the apical solution. Data were normalised by subtraction of the steady-state current after amiloride addition prior to F508del-CFTR activation by forskolin. (**B** – **D**) Summary data show the magnitude of baseline I_sc_ and R_t_ before amiloride addition and the change in I_sc_ (ΔI_sc_) for the indicated conditions. Symbols represent individual values and lines are means ± SD (elexacaftor-tezacaftor-ivacaftor (ETI): +phosphate, n = 9; –phosphate, n = 9).

Our results demonstrate that extracellular phosphate noticeably improved the function of corrector-rescued F508del-CFTR. To investigate whether this effect of phosphate involves a sodium-dependent phosphate transporter, we repeated the elexacaftor-tezacaftor-ivacaftor experiments in the absence of sodium in the solution bathing the apical membrane (Fig. 5). As expected, the removal of sodium from the apical bathing solution greatly reduced baseline I_sc_ (compare Fig. 4C with Fig. 5B). Importantly, in the absence of extracellular sodium, but in the presence of phosphate, forskolin no longer augmented elexacaftor-tezacaftor-ivacaftor-rescued F508del-CFTR function (Fig. 5D). This result demonstrates that the modulatory effect of phosphate is sodium dependent.

### 3.6 Acute addition of phosphate is sufficient to enhance the function of F508del-CFTR after correction by elexacaftor-tezacaftor-ivacaftor

For the studies described thus far, the effect of extracellular phosphate was tested by removing all phosphate from the recording solutions and comparing responses to epithelia with phosphate present throughout. To investigate the kinetics of the response to phosphate, elexacaftor-tezacaftor-ivacaftor-treated CF hAEC epithelia were mounted in Ussing chambers and bathed in phosphate-free solutions. After exposure to amiloride, phosphate was added directly to the phosphate-free solution bathing the apical membrane. The total magnitude of the acute F508del-CFTR-mediated I_sc_ was determined (~10 min after phosphate addition) and compared to results obtained when phosphate was present for the entire experiment (~50 min total time), with each condition studied simultaneously (Supplementary Fig. 6). The acute addition of phosphate caused a rapid increase in I_sc_ which stabilised within 1-2 min (Supplementary Fig. 6A). Importantly, the total magnitude of F508del-CFTR-mediated I_sc_ was identical in epithelia that were acutely exposed to phosphate, compared to those epithelia that had phosphate present throughout the experiment (Supplementary Fig. 6D). These results demonstrate that a relatively short exposure to phosphate is sufficient to improve the activity of F508del-CFTR rescued by elexacaftor-tezacaftor-ivacaftor.

## 4 Discussion

This study demonstrates that the activity of CFTR corrector-rescued F508del-CFTR was dependent on extracellular phosphate. This novel effect of phosphate was observed in FRT epithelia heterologously expressing F508del-CFTR and fully differentiated human airway epithelial cells (genotype: F508del/F508del), suggesting that it was independent of cell context. However, extracellular phosphate was without effect on low temperature-rescued F508del-CFTR and wild-type CFTR, suggesting that the response to phosphate might be specific for mutant CFTR whose trafficking (folding) defects were rescued by the CFTR correctors VX-809, C18 and elexacaftor-tezacaftor-ivacaftor [9,23,25,26].

Under the experimental conditions used, the effect of extracellular phosphate on CFTR corrector-rescued F508del-CFTR might result from three possible mechanisms: (i) increasing the number of channels in the apical membrane by either stimulating trafficking/insertion of F508del-CFTR into the membrane or decreasing its retrieval to enhance channel stability; (ii) acting as a potentiator, thereby increasing the open probability (P_o_) of F508del-CFTR and (iii) augmenting anion flow through individual F508del-CFTR Cl^−^ channels. However, these three mechanisms are not mutually exclusive and more than one might be involved.

For two reasons, the response to phosphate is unlikely to be explained by the recruitment of F508del-CFTR Cl^−^ channels to the apical membrane or enhancing their plasma membrane stability. First, acute addition of phosphate to the solution bathing the apical membrane increased CFTR-mediated Cl^−^ currents by a comparable amount as sustained exposure (Supplementary Fig. 6). Second, phosphate was present in the culture medium and was only absent from control experiments for the duration of Ussing chamber recordings. The fact that the response to acute addition of phosphate was rapid suggests an effect on channel gating and, hence P_o_. CFTR activity is tightly regulated to control the hydration and pH of epithelial secretions [28]. The channel is primarily activated by protein kinase A (PKA)-dependent phosphorylation of its regulatory domain (RD) [29]. Then, cycles of ATP binding and hydrolysis at two ATP-binding sites located at the interface of the nucleotide-binding domain (NBD) dimer controls channel gating and therefore, P_o_ [29]. Among the factors that modulate channel gating are the products of ATP hydrolysis, ADP and phosphate. ADP competitively inhibits CFTR gating with its major effect at ATP-binding site 2 [29,30]. By contrast, in the presence of ATP, raising the cytosolic phosphate concentration increased the P_o_ of wild-type CFTR by accelerating channel opening [22]. Phosphate did not change anion flow through individual CFTR Cl^−^ channels and was without effect on the number of active channels [22]. Because phosphate stimulated a CFTR construct lacking most of the RD (ΔR-S660A-CFTR) and was without effect on unphosphorylated wild-type CFTR [22], its enhancement of channel gating likely reflects an effect at ATP-binding site 2. Thus, extracellular phosphate likely enhances the function of CFTR corrector-rescued F508del-CFTR by modifying channel gating.

A plausible explanation for why extracellular phosphate was without effect on wild-type CFTR is the gating defect of F508del-CFTR [1]. Under the experimental conditions used, insufficient phosphate was likely transported into cells to noticeably increase the already high P_o_ of wild-type CFTR and hence, the magnitude of CFTR-mediated Cl^−^ current. Because the single-channel behaviour of F508del-CFTR is equivalent after rescue by either VX-809 or low temperature [31], a similar explanation does not account for the different effects of extracellular phosphate after F508del-CFTR correction by these treatments. Instead, the lack of effect of extracellular phosphate on low temperature-rescued F508del-CFTR might be explained by the more extensive correction of the mutant protein by low temperature than individual CFTR correctors [24], leading to greater plasma membrane expression with low temperature. Although this idea is supported by greater F508del-CFTR function in FRT epithelia heterologously expressing F508del-CFTR incubated at low temperature than rescued with either VX-809 or C18, it is not supported by the effect of phosphate on the function of native F508del-CFTR rescued by elexacaftor-tezacaftor-ivacaftor. Like the action of low temperature [24], elexacaftor and tezacaftor robustly reverse F508del-CFTR misfolding [27]. Future studies should therefore directly compare the effect of extracellular phosphate on F508del-CFTR rescued by either low temperature or elexacaftor-tezacaftor-ivacaftor.

For extracellular phosphate to modulate CFTR channel gating at its NBDs, transmembrane phosphate transport is required. Previous work demonstrates that the sodium-dependent phosphate transporter SLC34A2 is expressed in the respiratory airways [19]. Building on these data, we showed that SLC34A2 expression is equivalent in CF and non-CF human airway epithelial cells, and that the modulatory effect of phosphate required extracellular sodium. However, the present results do not exclude the participation of other sodium-dependent phosphate transporters. Although expression of the closely related phosphate transporters SLC34A1 and SLC3A3 is largely restricted to the renal proximal tubule, SLC20 transporters are ubiquitously expressed at the mRNA level and SLC17A2 is found in the lung [32,33]. To understand better the role of SLC34A2 and other phosphate transporters in airway epithelia will require the development of specific pharmacological tools and/or the manipulation of transporter gene expression.

The requirement for extracellular phosphate, likely mediated by SLC34A2, observed in the present study is comparable to that recently described for SLC6A14 [16]. Uptake of luminal L-Arginine by SLC6A14 led to the stimulation of CFTR function, rather than alteration of CFTR expression in the plasma membrane [16]. However, and in contrast to our results, L-Arginine also stimulated ‘resting’ CFTR function (after correction), in the absence of PKA and CFTR potentiators [16]. Consistent with previous results [22], in the present study extracellular phosphate was without effect on the ‘resting’ activity of F508del-CFTR after its rescue by CFTR correctors (Figs. 3C and 4C).

## 5 Conclusion

The phosphate transporter SLC34A2 is expressed in human airway epithelial cells and its relative abundance is similar in CF and non-CF cells. Luminal phosphate stimulates the activity of F508del-CFTR rescued by the CFTR correctors VX-809, C18 or VX-445+VX-661 with VX-770 and its action was sodium-dependent. These and other data [20], [21], [22] suggest that *in vivo*, SLC34A2 function regulates both the amount of phosphate in ASL and the local cytosolic concentration of phosphate, thereby enhancing the function of CFTR corrector-rescued F508del-CFTR by altering channel gating. Since mutations in SLC34A2 which reduce phosphate transport are linked to lung disease [19], [20], [21], we speculate that any SNPs which alter SLC34A2 activity might indirectly affect F508del-CFTR function and potentially impact the outcome of CFTR corrector therapies.

## Credit author statement

Vinciane Saint-Criq: Conceptualization, Methodology, Investigation, Formal analysis, Data Curation, Visualization, Writing – Review and Editing. Yiting Wang: Investigation, Validation, Formal analysis, Visualization. Livia Delpiano: Investigation, Formal analysis, Visualization. JinHeng Lin: Investigation, Formal analysis. David N. Sheppard: Methodology, Validation, Writing – Review and Editing, Supervision, Funding. Michael A. Gray: Conceptualization, Methodology, Writing – Original Draft, Writing – Review and Editing, Supervision, Project Administration, Funding. All authors approved the final version of the manuscript.

## Funding

This work was supported by a CF Trust Strategic Research Centre award (SRC013) and a Medical Research Council (MRC) Confidence in Concept award (MC_PC_15030) (MAG) and the CF Trust (DNS). Cells from Dr. Randell were supported by Cystic Fibrosis Foundation grant BOUCHE15R0 and NIH grant P30DK065988. Cells from Dr. Pedemonte were supported by the Italian Ministry of Health through Cinque per Mille and Ricerca Corrente (Linea 1).

## Declaration of Competing Interest

The authors declare that they have no conflicts of interest with the contents of this manuscript.

## Appendix Supplementary materials

Image, application 1

Image, image 2

Image, image 3

Image, image 4

Image, image 5

Image, image 6

Image, application 1

## Footnotes

Supplementary material associated with this article can be found, in the online version, at doi:10.1016/j.jcf.2021.04.013.
