# Supplementary figures and images for: Extracellular phosphate enhances the function of F508del-CFTR rescued by CFTR correctors
Source: J Cyst Fibros. 2021 Sep;20(5):843–50. doi: 10.1016/j.jcf.2021.04.013 (PMC8503924; doi:10.1016/j.jcf.2021.04.013)

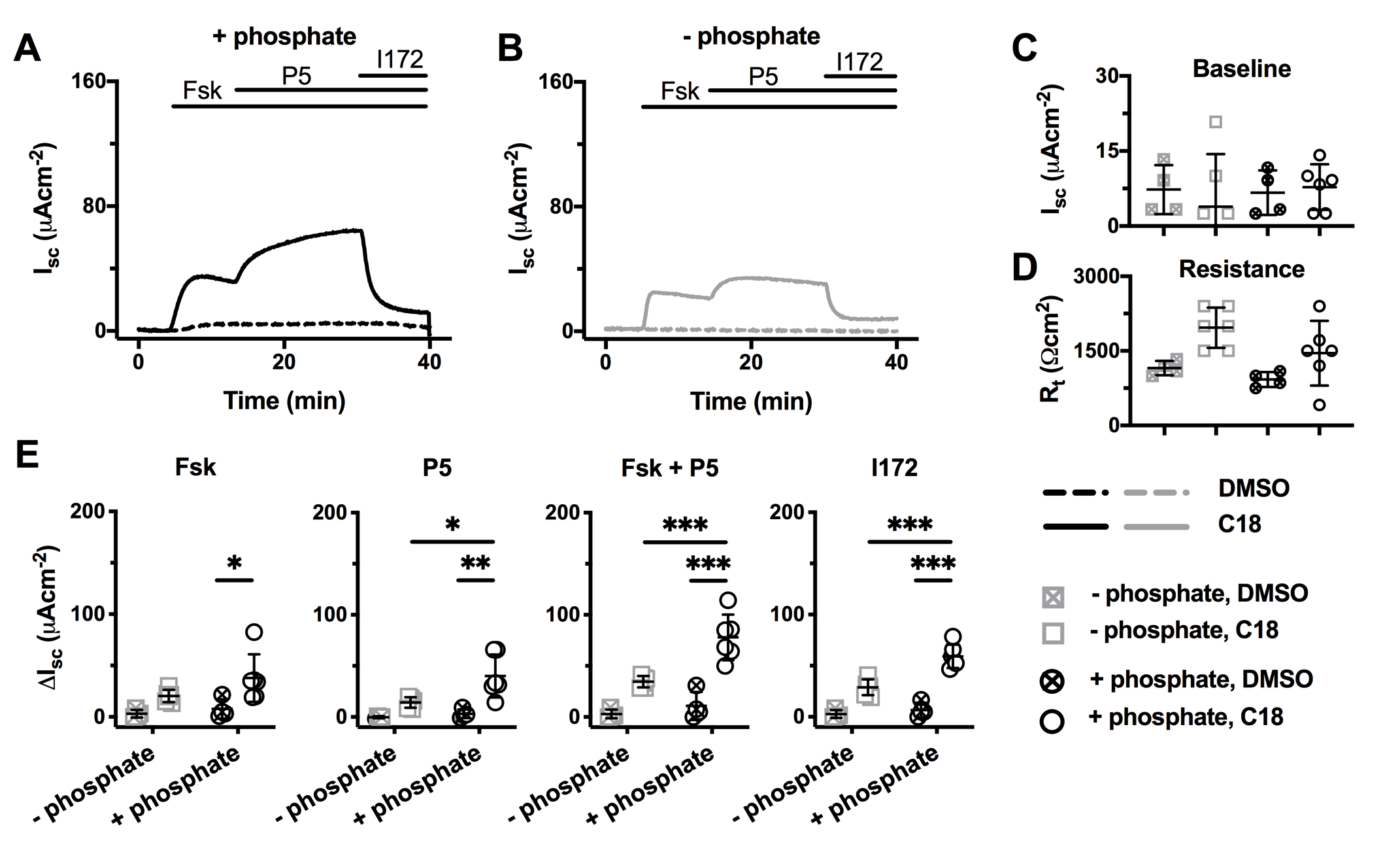

Supplement: Supplementary file 1 [file mmc1.zip › mmc1.tiff]

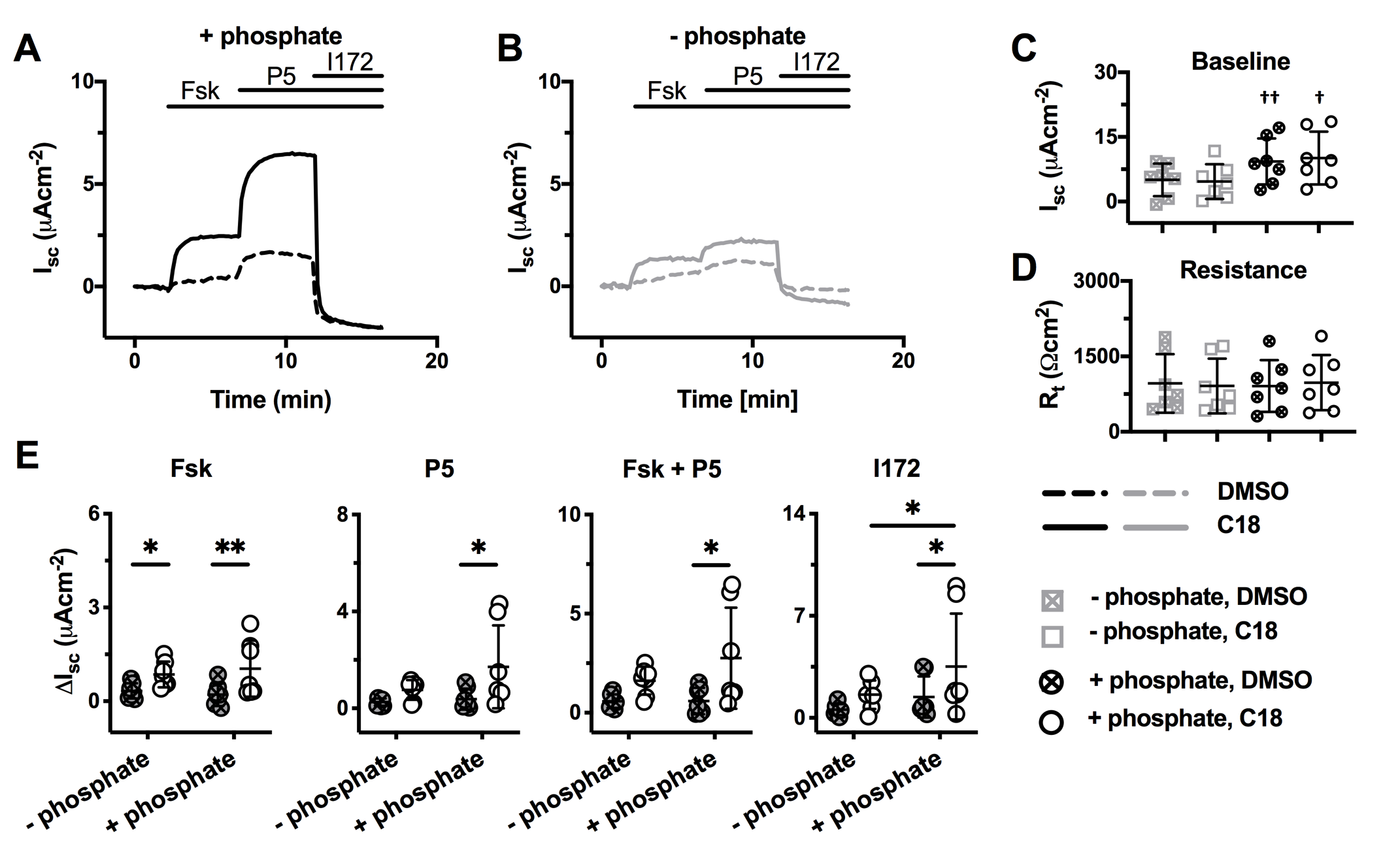

Supplement: Supplementary file 2 [file mmc2.zip › mmc2.tiff]

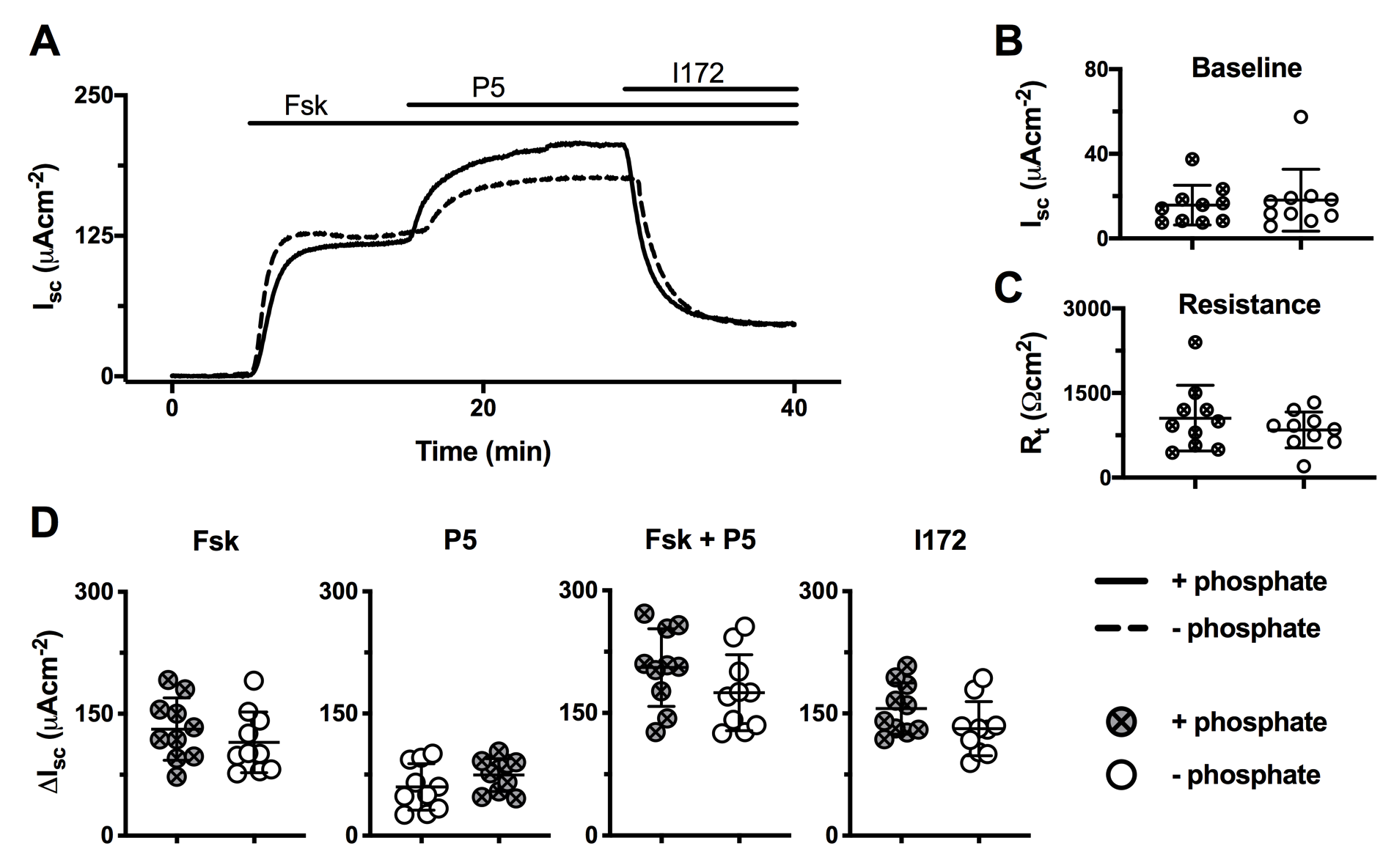

Supplement: Supplementary file 3 [file mmc3.zip › mmc3.tiff]

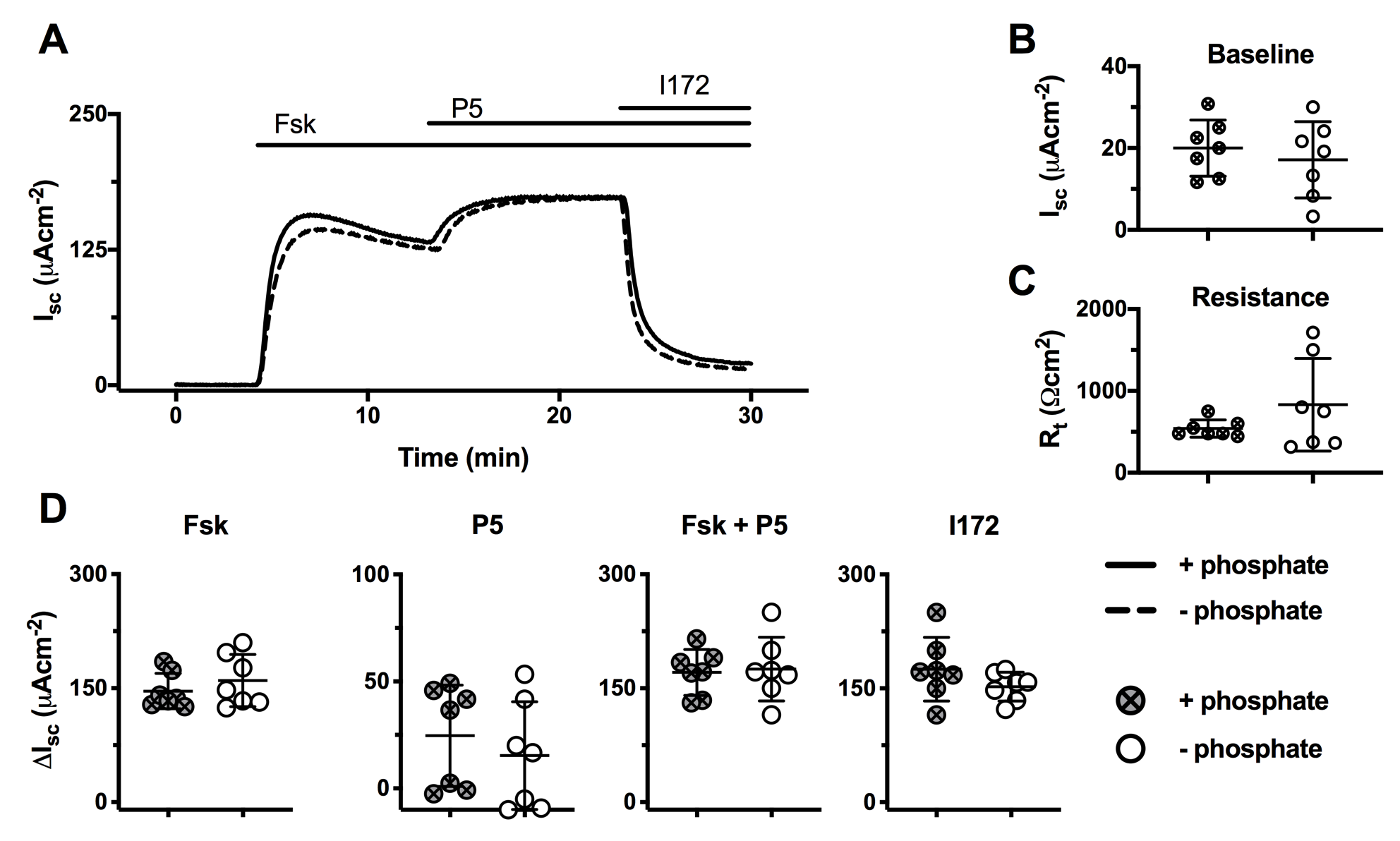

Supplement: Supplementary file 4 [file mmc4.zip › mmc4.tiff]

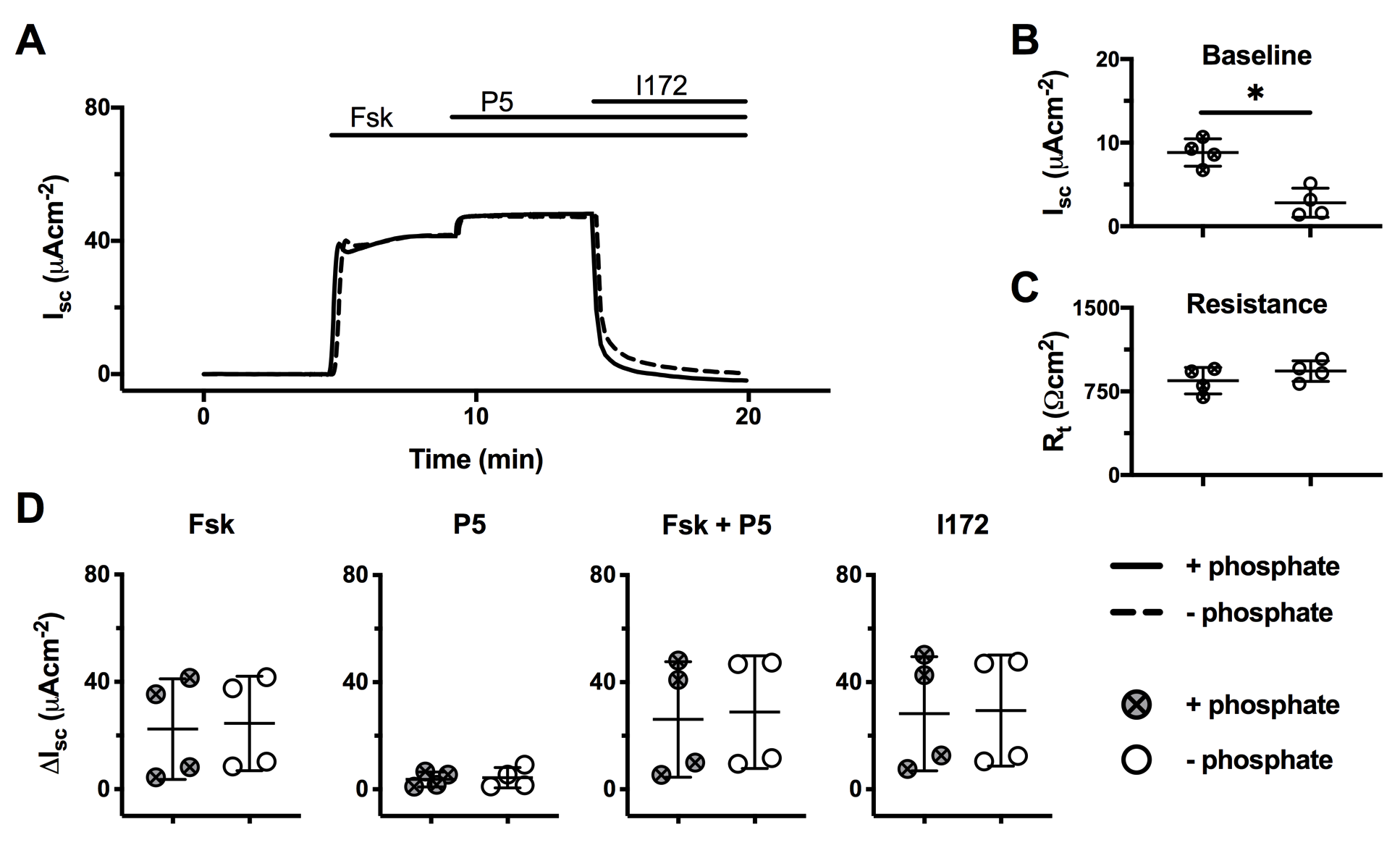

Supplement: Supplementary file 5 [file mmc5.zip › mmc5.tiff]

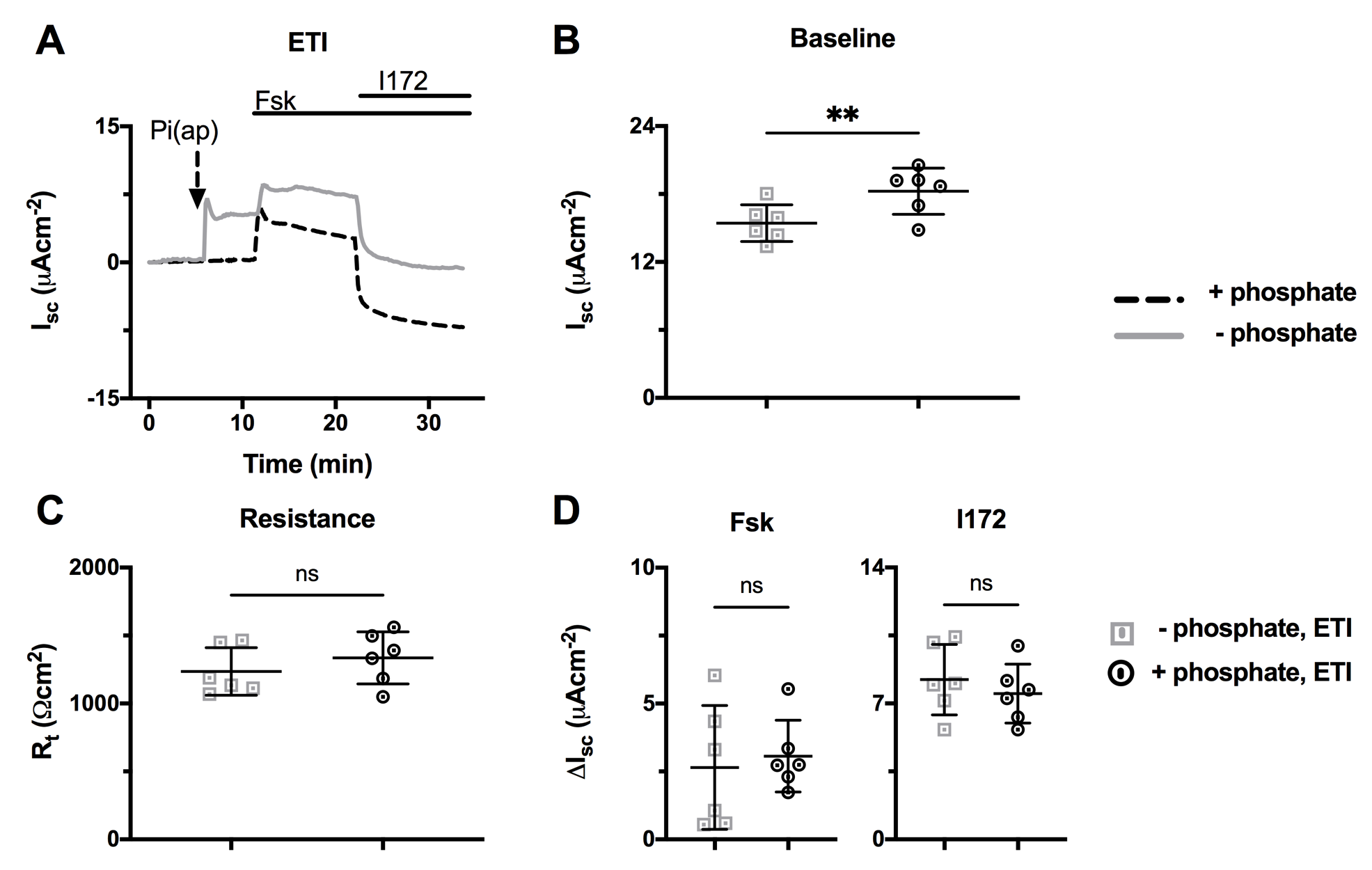

Supplement: Supplementary file 6 [file mmc6.zip › mmc6.tiff]
