# Supplementary material for: Extracellular phosphate enhances the function of F508del-CFTR rescued by CFTR correctors
Source: J Cyst Fibros. 2021 Sep;20(5):843–50. doi: 10.1016/j.jcf.2021.04.013 (PMC8503924; doi:10.1016/j.jcf.2021.04.013)
Supplement: Supplementary file 7 [file mmc7.docx]

**SUPPLEMENTARY MATERIAL**

**Methods**

**Cells and cell culture**

Primary non-CF (n = 3 donors) and CF (n = 3 donors, all F580del/F508del genotype) human Airway Epithelial Cells (hAECs) were a kind gift from S. H. Randell (University of North Carolina (UNC) CF Center Tissue Procurement and Cell Culture Core). They were obtained under protocol #03-1396 approved by the UNC Biomedical Institutional Review Board. All hAECs were first expanded using the Conditionally Reprogrammed Cell (CRC) culture method [1]. In brief, hAECs were seeded onto 3T3J2 fibroblasts, inactivated with mitomycin C (4 µg/ml, 2 h, 37 °C) and grown in medium containing the ROCK inhibitor Y-27632 (10 μM) until 80% confluent. Cells then underwent double trypsinisation to first remove the fibroblasts and then detach the hAECs. At this stage, the hAECS were counted and frozen down. After thawing, cryopreserved hAECs were seeded directly onto human placental collagen (HPC, Sigma-Aldrich, C8374)-coated semi-permeable supports (Costar Transwells, 6.5 mm) in bilateral differentiating medium [2]. After they reached confluence (3-4 days), the apical medium was removed and the hAECs were fully differentiated under Air-Liquid Interface (ALI) conditions. Ciliogenesis started approximately 12-15 days after seeding and the cells were used for experiments between days 25 and 35 after seeding.

Fischer Rat Thyroid (FRT) epithelial cells heterologously expressing wild-type (WT)- and F508del-CFTR were a kind gift of N. Pedemonte, Instituto Giannina Gaslini) and LJV Galietta (Telethon Institute of Genetics and Medicine). FRT cells were grown as previously described [3]. In brief, cells were expanded in Coon’s modified Ham’s F-12 medium (Sigma Aldrich F6636) supplemented with 10% FBS, 2% glutamine, 50 U ml^−1^ penicillin, 50 μg ml^−1^ streptomycin and zeocin (600 μg ml^−1^) as the selection agent. Once cells reached 80% confluence, they were trypsinised and seeded onto semi-permeable supports (Costar Transwells, 6.5 mm) at a density of 7.5×10^5^ cells per cm^2^. Apical and basolateral media were replaced every 48 h and cells were used 6-8 days after seeding.

**RNA extraction, PCR and real-time quantitative PCR analysis**

RNA isolation from fully differentiated hAECs and FRT cells was performed using PureLink^®^ RNA Mini Kit (12183018A, Ambion, Life Technologies) following the manufacturer’s instructions. In brief, lysates were mixed with 70% ethanol and loaded onto a silica-membrane column. Columns were washed and total RNA was eluted in nuclease-free water and stored at –80 °C until use. Prior to reverse transcription, DNase treatment was performed on 300 ng RNA using RNAse-free DNAse I (04716728001, Roche) at 37 °C for 10 min. The reaction was stopped by increasing the temperature to 70 °C for 10 min. Complementary DNA (cDNA) was synthesized from DNase I-treated total RNA (300 ng) using M-MLV Reverse Transcriptase (Promega) as per the supplier’s protocol (1 h at 37 °C followed by 10 min at 70 °C).

Polymerase Chain Reaction (PCR) was performed in a total volume of 25 μl using 2 μl cDNA, 5 µl 5X Q5 reaction buffer, 0.5 µl 10 mM dNTP, 1.25 µl 10 μM forward primer, 1.25 µl 10 μM reverse primer, 0.25 µl Q5 high fidelity polymerase and 5 µl 5X Q5 high enhancer. The sequences for the primer pairs were (human *SLC34A2*, forward: ATCAAGAAGACCATCAACACT, reverse: GTTATCACGCCGATTCCAA, predicted product length = 155 bp; rat *slc34a2*, forward ACGACGTGTTTGGCACATTG, reverse: GCCTGGGACACAGGTCTTAC; predicted product length = 109 bp). The expression of 18S rRNA was used as internal control (forward: CTCTAGATAACCTCGGGCCG, reverse: GTCGGGAGTGGGTAATTTGC, predicted product length = 209 bp in human cells, 208 bp in rat cells). For both genes, the PCR protocol was as follows: initial denaturation was performed at 98 °C for 30 s followed by 35 cycles at 98 °C for 5 s, 70 °C for 30 s and 72 °C for 20 s. After the 35^th^ cycle, the final extension phase was performed at 72 °C for 2 min and PCR products were then kept at 4 °C until loaded onto a SYBR Green-containing agarose gel (2 % in 1X Tris-borate-EDTA-TBE). Electrophoresis lasted for 90 min at 100 V and PCR products were visualized using the Fuji LAS-3000 Image reader.

Real-time quantitative PCR (qPCR) was performed in a total volume of 15 μl using 2x LightCycler^®^ 480 SYBR Green I Master (Roche, 04707516001), 1.5 μl cDNA, 0.2 μM forward primer and 0.2 μM reverse primer in a 96-well plate (same primer pairs as for classical PCR). The PCR was run using the standard program: 95 °C 10 min, 40 times of cycling 95 °C 15 s and 60 °C 1 min in a 96-well plate. Results are shown as Relative Quantity (RQ) of mRNA copies compared to the determined control condition, calculated using the 2^-ΔΔCt^ method [4].

**Short‐circuit current measurements in Ussing chambers**

*Human airway epithelial cells endogenously expressing CFTR*: Cells grown on Costar 6.5 mm inserts were mounted in the EasyMount Ussing Chamber System (VCC MC8, Physiologic Instrument Inc, USA) and bathed in a basolateral HCO_3_^-^ Krebs Ringer Buffer (KRB) and apical low Cl^-^ KRB, in the presence or absence of phosphate (K_2_HPO_4_ and KH_2_PO_4_), continuously gassed with 95% O_2_-5% CO_2_ and maintained at 37 °C. The basolateral solution contained (in mM) 115 NaCl, 25 NaHCO_3_, 1.2 MgCl_2_, 1.2 CaCl_2_, 5 Glucose (pH 7.4) and the apical solution contained (in mM) 115 Na-Gluconate, 25 NaHCO_3_, 1.2 MgCl_2_, 5 Glucose (pH 7.4). Solutions with phosphate, contained (in mM) 2.4 KH_2_PO_4_, 1.24 K_2_HPO_4_, and for the apical solution NaCl was reduced to 1.2 mM and CaCl_2_ was increased to 4.0 mM. Solutions without phosphate, contained 5 mM KCl, and the apical solution additionally contained (in mM) 1.2 CaCl_2_ and 2.8 Ca-gluconate. For sodium-free solutions, sodium salts were substituted with the same concentration of NMDG-Cl, NMDG-gluconate and choline-HCO_3_.

Epithelia were voltage-clamped at 0 mV and left to equilibrate for at least 20 min before ion transport studies were performed by the sequential and cumulative addition of amiloride (10 μM), forskolin (10 μM), P5 (10 μM) and CFTR_inh_-172 (20 μM); for elexacaftor-tezacaftor-ivacaftor-treated cells, P5 was omitted. All compounds were added to the solution bathing the apical membrane with the exception of forskolin which was added to both the apical and basolateral solutions. Changes in short-circuit current (ΔI_sc_) were monitored using Ag/AgCl reference electrodes. The transepithelial short-circuit current (I_sc_) and the transepithelial resistance (R_t_) were recorded using Ag-AgCl electrodes in 3 M KCl agar bridges [5], and results normalized to an area of 1 cm^2^ and expressed as I_sc_ (µA.cm^−2^) with the Acquire & Analyze software (Physiologic Instruments).

*FRT epithelia heterologously expressing CFTR*: CFTR-mediated transepithelial Cl^-^ currents were recorded using a modification of the method described by Meng et al. [6]. FRT epithelia were mounted in modified Ussing chambers (Warner Instrument Corp., Dual Channel chamber) and bathed in the same solutions used to study hAECs (see above) to magnify CFTR-mediated transepithelial Cl^-^ currents without permeabilising the basolateral membrane. All solutions were maintained at 37 °C and bubbled continuously with 5% CO_2_.

After cancelling voltage offsets, transepithelial voltage (referenced to the basolateral solution) was clamped at 0 mV and I_sc_ recorded continuously using an epithelial voltage-clamp amplifier (Warner Instrument Corp., model EC-825), digitizing data as described previously [7]. Except for the omission of amiloride, the same protocol used to record CFTR-mediated transepithelial Cl^-^ currents in hAECs was employed to study FRT epithelia. Under these experimental conditions, flow of current from the basolateral to the apical solution corresponds to Cl^-^ movement through open CFTR Cl^-^ channels and is shown as an upward deflection. The resistance of the filter and solutions in the absence of cells was subtracted from all measurements. For the purpose of illustrating I_sc_ time courses, file sizes were compressed by 100-fold data reduction.

**Reagents**

The CFTR modulators lumacaftor, tezacaftor and ivacaftor were purchased from Selleck Chemicals, while the CFTR corrector elexacaftor was purchased from MedChemExpress. The CFTR corrector C18 and the CFTR potentiator P5 were generous gifts of the Cystic Fibrosis Foundation CFTR Chemical compound Distribution Program administered by RJ Bridges (Rosalind Franklin University of Medicine and Science). All other chemicals, including forskolin and CFTR_inh_-172 were of reagent grade and supplied by Sigma-Aldrich Ltd. (now Merck Life Science UK Ltd.).

Forskolin was dissolved in methanol. All other reagents were solubilised in DMSO before storage at –80 °C. To achieve final concentrations, stock solutions were diluted in KRB. DMSO was without effect on CFTR activity [8]. On completion of experiments, Ussing chambers were thoroughly cleaned before re-use [6].

**Statistical analysis**

Results are expressed as means ± SD of n observations. For non-parametric data, the Wilcoxon signed rank test (paired) and Mann-Whitney rank sum test (unpaired) were used. For multiple sets of data, one-way and two-way analysis of variance (ANOVA) followed by Tukey’s or Sidak’s post-tests were employed, where appropriate. Differences were considered statistically significant when P < 0.05. Tests were performed using either Prism (version 8, GraphPad Software) or SigmaPlot^TM^ (version 13.0, Systat Software Inc.).

**References**

[1] Suprynowicz FA, Upadhyay G, Krawczyk E, Kramer SC, Hebert JD, Liu X, et al. Conditionally reprogrammed cells represent a stem-like state of adult epithelial cells. Proc Natl Acad Sci U S A 2012;109:20035–40. https://doi.org/10.1073/pnas.1213241109.

[2] Randell SH, Fulcher ML, O’Neal W, Olsen JC. Primary epithelial cell models for cystic fibrosis research. Methods Mol Biol 2011;742:285–310. https://doi.org/10.1007/978-1-61779-120-8_18.

[3] Zegarra-Moran O, Romio L, Folli C, Caci E, Becq F, Vierfond J-M, et al. Correction of G551D-CFTR transport defect in epithelial monolayers by genistein but not by CPX or MPB-07. Br J Pharmacol 2002;137:504–12. https://doi.org/10.1038/sj.bjp.0704882.

[4] Livak KJ, Schmittgen TD. Analysis of relative gene expression data using real-time quantitative PCR and the 2^–ΔΔCT^ method. Methods 2001;25:402–8. https://doi.org/10.1006/meth.2001.1262.

[5] Saint-Criq V, Kim SH, Katzenellenbogen JA, Harvey BJ. Non-genomic estrogen regulation of ion transport and airway surface liquid dynamics in cystic fibrosis bronchial epithelium. PLoS One 2013;8:e78593. https://doi.org/10.1371/journal.pone.0078593.

[6] Meng X, Wang Y, Wang X, Wrennall JA, Rimington TL, Li H, et al. Two small molecules restore stability to a subpopulation of the cystic fibrosis transmembrane conductance regulator with the predominant disease-causing mutation. J Biol Chem 2017;292:3706–19. https://doi.org/10.1074/jbc.M116.751537.

[7] Li H, Findlay IA, Sheppard DN. The relationship between cell proliferation, Cl^-^ secretion, and renal cyst growth: a study using CFTR inhibitors. Kidney Int 2004;66:1926–38. https://doi.org/10.1111/j.1523-1755.2004.00967.x.

[8] Schmidt A, Hughes LK, Cai Z, Mendes F, Li H, Sheppard DN, et al. Prolonged treatment of cells with genistein modulates the expression and function of the cystic fibrosis transmembrane conductance regulator. Br J Pharmacol 2008;153:1311–23. https://doi.org/10.1038/sj.bjp.0707663.

**Figure Legends**

**Supplementary Figure 1: Phosphate enhances C18-rescued F508del-CFTR-mediated Cl^-^ currents in FRT epithelia.** (**A** and **B**) Representative I_sc_ recordings of C18-rescued F508del-CFTR in the presence (**A**) and absence (**B**) of phosphate (1.24 mM K_2_HPO_4_ and 2.4 mM KH_2_PO_4_) in the Krebs Ringer Buffer (KRB). Prior to study, F508del-CFTR-expressing FRT epithelia were treated with C18 (3 μM) or DMSO (0.1% v·v^-1^) for 48 h at 37 °C. At the indicated times, F508del-CFTR-mediated Cl^-^ currents were activated with forskolin (Fsk; 10 μM), potentiated with P5 (10 μM) and inhibited with CFTR_inh_-172 (I172; 20 μM); continuous lines indicate the presence of compounds in the apical solution only or the apical and basolateral solutions (forskolin) during I_sc_ recordings. Data were normalised by subtraction of the baseline current prior to F508del-CFTR activation by forskolin. (**C** – **E**) Summary data show the magnitude of baseline I_sc_, R_t_ before forskolin addition and the change in I_sc_ (ΔI_sc_) for the indicated conditions. Symbols represent individual values and lines are means ± SD (n = 6); *, P < 0.05; **, P < 0.01; ***, P < 0.001 (Two-way ANOVA with Tukey’s multiple comparison test).

**Supplementary Figure 2: Phosphate increases C18-rescued F508del-CFTR-mediated Cl^-^ currents in hAEC epithelia.** (**A** and **B**) Representative I_sc_ recordings of C18-rescued F508del-CFTR in the presence (**A**) and absence (**B**) of phosphate (1.24 mM K_2_HPO_4_ and 2.4 mM KH_2_PO_4_) in the KRB. Prior to study, hAEC epithelia (genotype: F508del/F508del) were treated with C18 (3 μM) or DMSO (0.1% v·v^-1^) for 48 h at 37 °C. At the indicated times, F508del-CFTR-mediated Cl^-^ currents were activated with forskolin (Fsk; 10 μM), potentiated with P5 (10 μM) and inhibited with CFTR_inh_-172 (I172; 20 μM); continuous lines indicate the presence of compounds in the apical solution only or the apical and basolateral solutions (forskolin) during I_sc_ recordings. Experiments were performed in the presence of amiloride (10 μM) in the apical solution. Data were normalised by subtraction of the steady-state current after amiloride addition prior to F508del-CFTR activation by forskolin. (**C** – **E**) Summary data show the magnitude of baseline I_sc_ and R_t_ before amiloride addition and the change in I_sc_ (ΔI_sc_) for the indicated conditions. Symbols represent individual values and lines are means ± SD (n = 5); *, P < 0.05; **, P < 0.01; ^†^, P < 0.05 vs. –phosphate; ^††^, P < 0.01 vs. –phosphate (Repeated Measure two-way ANOVA with Sidak’s multiple comparison test).

**Supplementary Figure 3: Phosphate fails to enhance low temperature-rescued F508del-CFTR-mediated Cl^-^ currents in FRT epithelia.** (**A**) Representative I_sc_ recordings of low temperature-rescued F508del-CFTR in the absence and presence of phosphate (1.24 mM K_2_HPO_4_ and 2.4 mM KH_2_PO_4_) in the KRB. Prior to study, F508del-CFTR-expressing FRT epithelia were incubated at 27 °C for 48 h. At the indicated times, F508del-CFTR-mediated Cl^-^ currents were activated with forskolin (Fsk; 10 μM), potentiated with P5 (10 μM) and inhibited with CFTR_inh_-172 (I172; 20 μM); continuous lines indicate the presence of compounds in the apical solution only or the apical and basolateral solutions (forskolin) during I_sc_ recordings. Data were normalised by subtraction of the baseline current prior to F508del-CFTR activation by forskolin. (**B** – **D**) Summary data show the magnitude of baseline I_sc_, R_t_ before forskolin addition and the change in I_sc_ (ΔI_sc_) for the indicated conditions. Symbols represent individual values and lines are means ± SD (n = 10).

**Supplementary Figure 4: Phosphate is without effect on WT-CFTR-mediated Cl^-^ currents in FRT epithelia.** (**A**) Representative I_sc_ recordings of WT-CFTR in the absence and presence of phosphate (1.24 mM K_2_HPO_4_ and 2.4 mM KH_2_PO_4_) in the KRB. At the indicated times, WT-CFTR-mediated Cl^-^ currents in FRT epithelia were activated with forskolin (Fsk; 10 µM), potentiated with P5 (10 µM) and inhibited with CFTR_inh_-172 (I172; 20 µM); continuous lines indicate the presence of compounds in the apical solution only or the apical and basolateral solutions (forskolin) during I_sc_ recordings. Data were normalised by subtraction of the baseline current prior to WT-CFTR activation by forskolin. (**B** – **D**) Summary data show the magnitude of baseline I_sc_, R_t_ before forskolin addition and the change in I_sc_ (ΔI_sc_) for the indicated conditions. Symbols represent individual values and lines are means ± SD (n = 7).

**Supplementary Figure 5: Phosphate fails to change WT-CFTR-mediated Cl^-^ currents in hAEC epithelia.** (**A**) Representative I_sc_ recordings of WT-CFTR in the absence and presence of phosphate (1.24 mM K_2_HPO_4_ and 2.4 mM KH_2_PO_4_) in the KRB. At the indicated times, WT-CFTR-mediated Cl^-^ currents in hAEC epithelia were activated with forskolin (Fsk; 10 μM), potentiated with P5 (10 μM) and inhibited with CFTR_inh_-172 (I172; 20 μM); continuous lines indicate the presence of compounds in the apical solution only or the apical and basolateral solutions (forskolin) during I_sc_ recordings. Experiments were performed in the presence of amiloride (10 μM) in the apical solution. Data were normalised by subtraction of the steady-state current after amiloride addition prior to WT-CFTR activation by forskolin. (**B** – **D**) Summary data show the magnitude of baseline I_sc_, R_t_ before amiloride addition and the change in I_sc_ (ΔI_sc_) for the indicated conditions. Symbols represent individual values and lines are means ± SD (n = 4 from 2 different donors); *, P < 0.05 (Mann-Whitney rank sum test).

**Supplementary Figure 6: Acute exposure to apical phosphate enhances elexacaftor-tezacaftor-ivacaftor- rescued F508del-CFTR-mediated Cl^-^ currents in hAEC epithelia.** (**A**) Representative I_sc_ recordings of elexacaftor-tezacaftor-ivacaftor (ETI) -rescued F508del-CFTR in the absence or presence of phosphate (1.24 mM K_2_HPO_4_ and 2.4 mM KH_2_PO_4_) in the KRB. Prior to study, hAEC epithelia (genotype: F508del/F508del) were treated with VX-445 (2 µM), VX-661 (3 µM) and VX-770 (1 µM) or DMSO (0.06% v·v^-1^) for 24 h at 37 °C. At the indicated time, phosphate (1.24 mM K_2_HPO_4_ + 2.4 mM KH_2_PO_4_) was added acutely to the apical solution of hAEC epithelia bathed in phosphate-free KRB [Pi(ap); grey line] and then F508del-CFTR-mediated Cl^-^ currents were activated with forskolin (Fsk; 10 μM), and inhibited with CFTR_inh_-172 (I172; 20 μM); continuous lines indicate the presence of compounds in the apical solution only or the apical and basolateral solutions (forskolin) during I_sc_ recordings. Experiments were performed in the presence of amiloride (10 μM) in the apical solution. Data were normalised by subtraction of the steady-state current after amiloride addition prior to F508del-CFTR activation by forskolin. (**C** – **E**) Summary data show the magnitude of baseline I_sc_ and R_t_ before amiloride addition and the change in I_sc_ (ΔI_sc_) for the indicated conditions. Symbols represent individual values and lines are means ± SD (n = 6).
